# Supplementary material for: Phenotypic and Genomic Characterization of the Comune di Sicilia Goat: Towards the Conservation of an Endangered Local Breed
Source: Animals (Basel). 2023 Oct 13;13(20):3207. doi: 10.3390/ani13203207 (PMC10603724; doi:10.3390/ani13203207)
Supplement: Supplementary file 1 [file animals-13-03207-s001.zip › Figure S2.pdf]

Figure S2: admixture analysis for a number of clusters (K) ranging from 2 to 15 and representation of cross-validation values (c-v) for all the tested K.

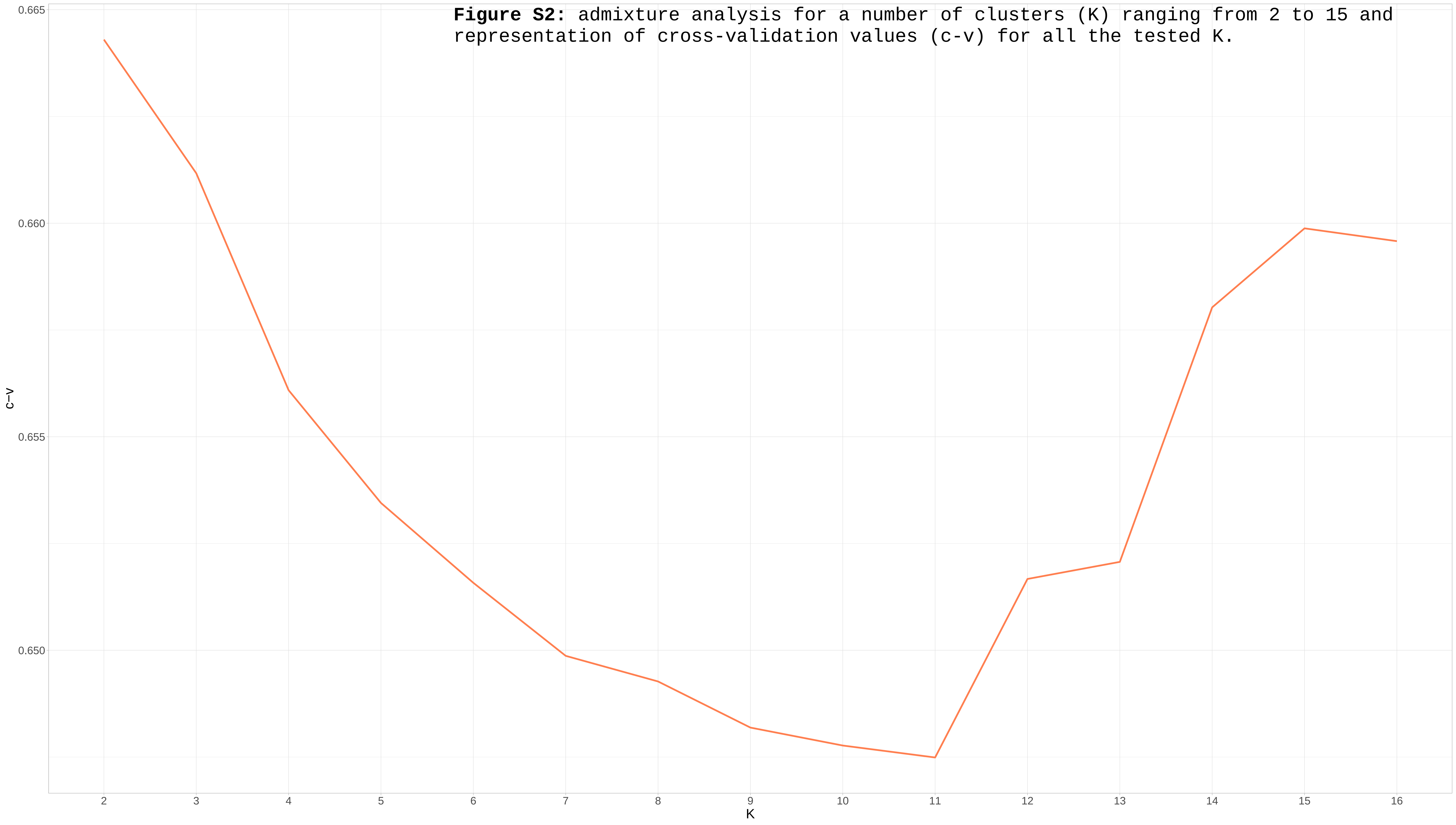

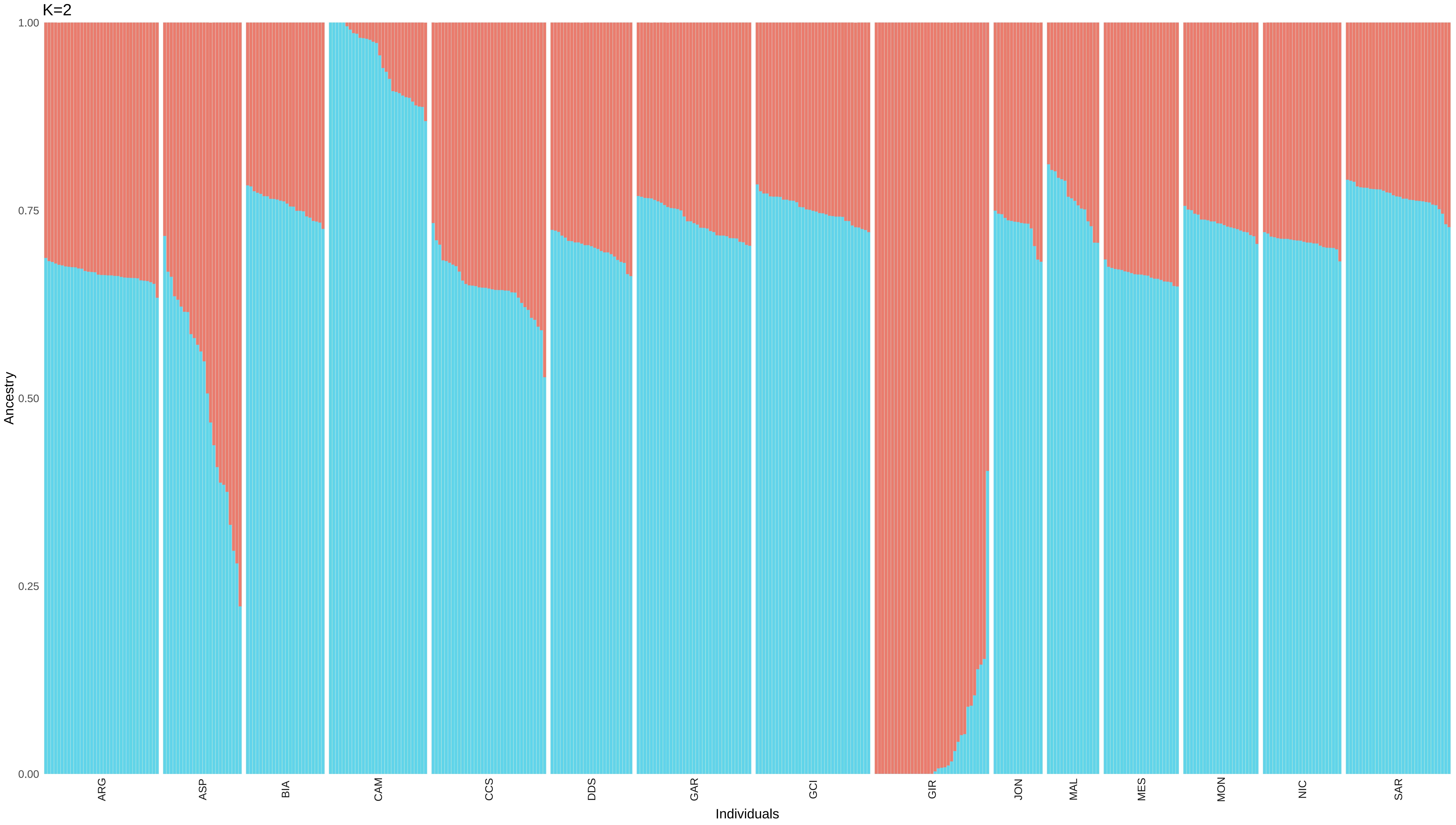

K=2
Ancestry
1.00
0.75
0.50
0.25
0.00
ARG
ASP
BIA
CAM
CCS
DDS
GAR
GCI
GIR
JON
MAL
MES
MON
NIC
SAR
Individuals

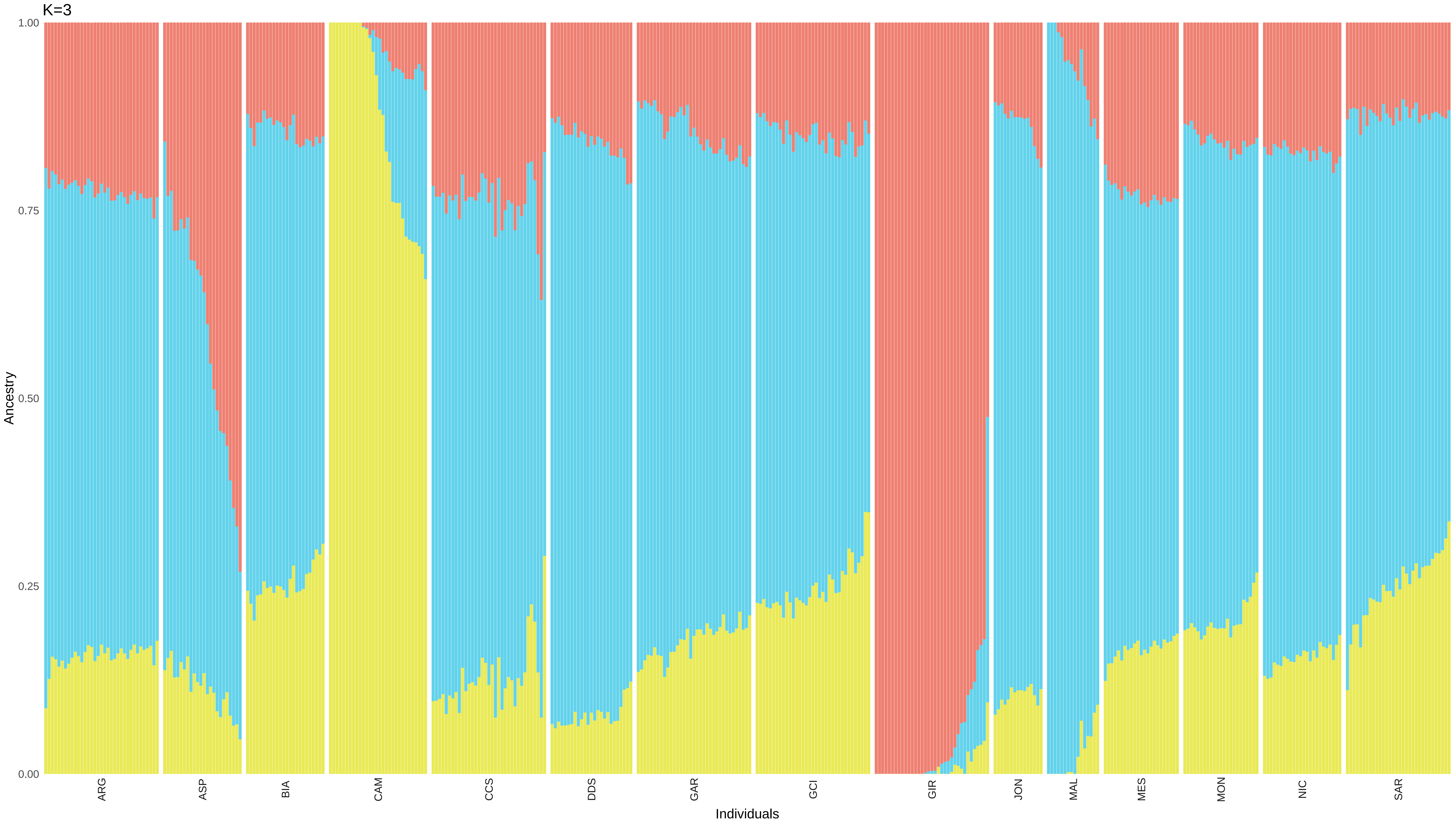
K=3
Ancestry
1.00
0.75
0.50
0.25
0.00
ARG
ASP
BIA
CAM
CCS
DDS
GAR
GCI
GIR
JON
MAL
MES
MON
NIC
SAR
Individuals

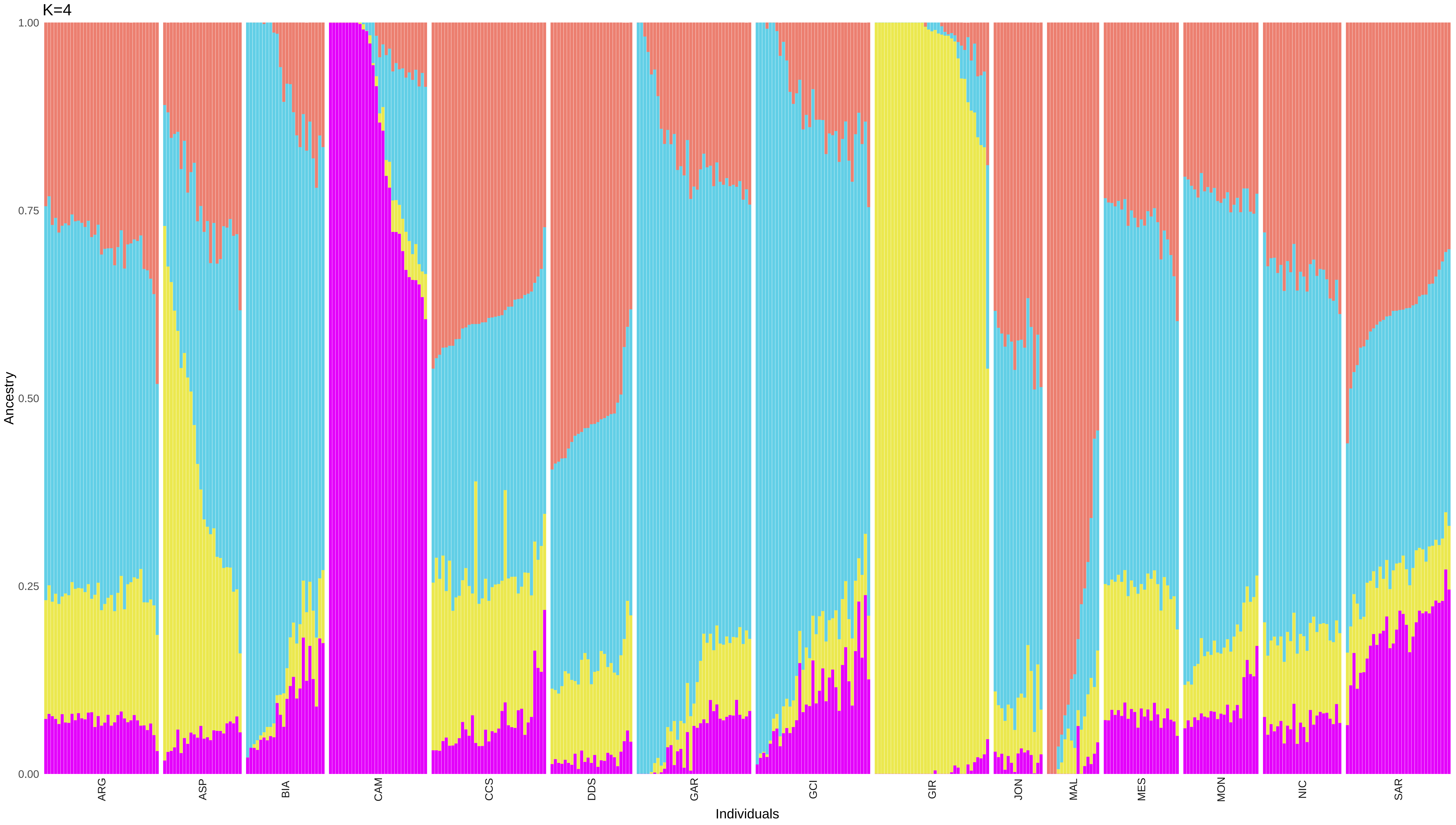
K=4
Ancestry
1.00
0.75
0.50
0.25
0.00
ARG
ASP
BIA
CAM
CCS
DDS
GAR
GCI
GIR
JON
MAL
MES
MON
NIC
SAR
Individuals

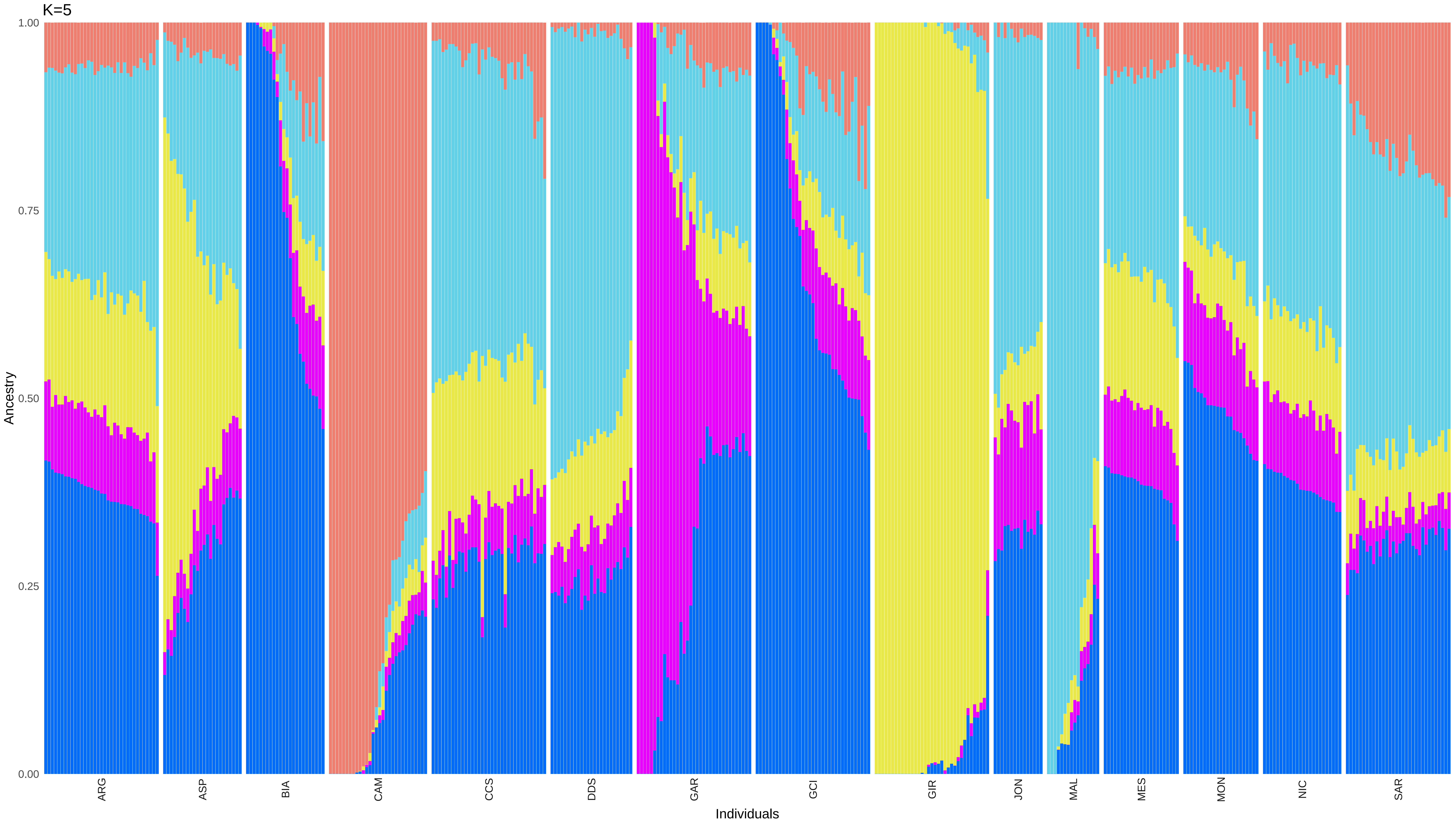
K=5
Ancestry
1.00
0.75
0.50
0.25
0.00
ARG
ASP
BIA
CAM
CCS
DDS
GAR
GCI
GIR
JON
MAL
MES
MON
NIC
SAR
Individuals

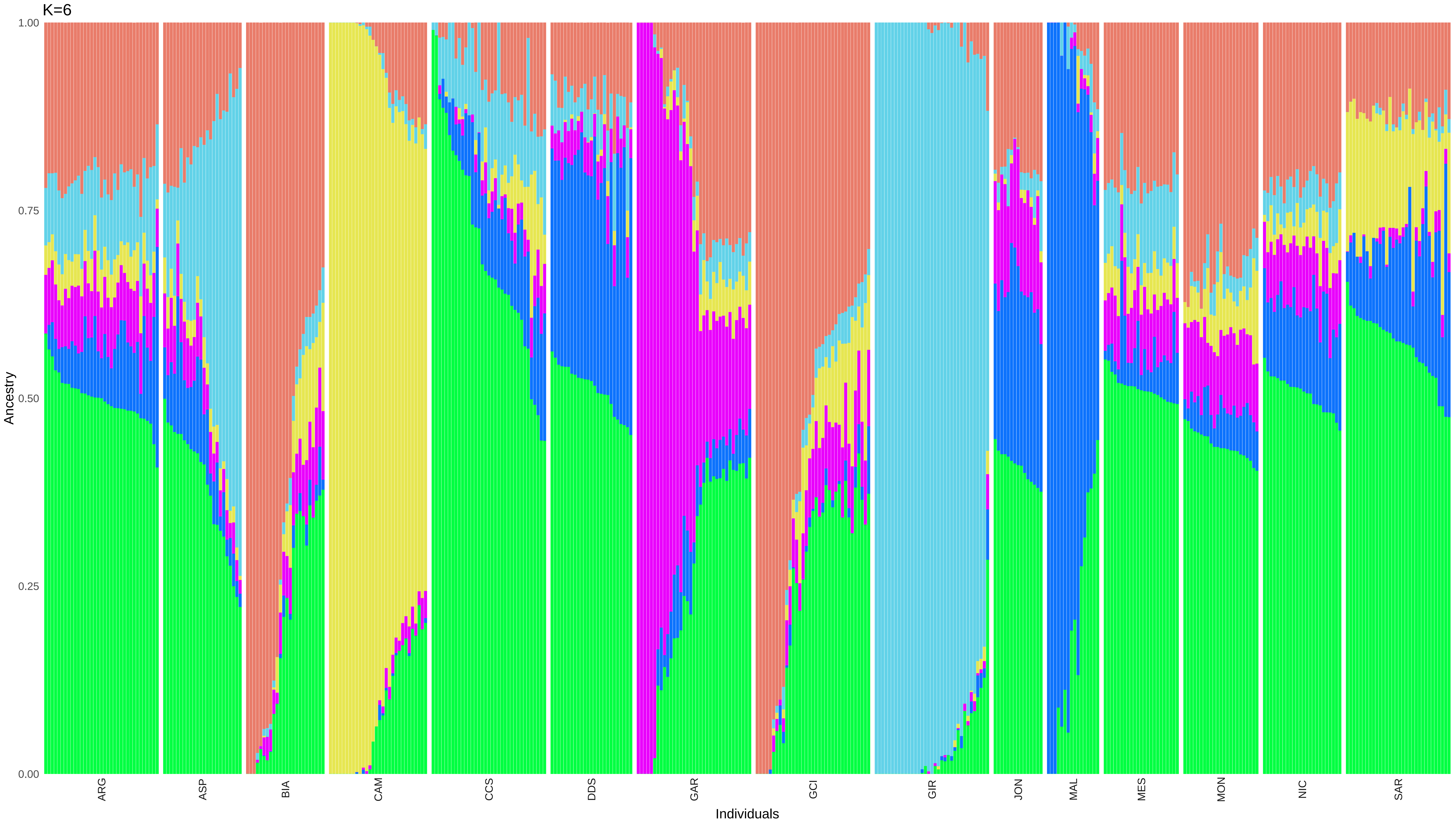
K=6
Ancestry
1.00
0.75
0.50
0.25
0.00
ARG
ASP
BIA
CAM
CCS
DDS
GAR
GCI
GIR
JON
MAL
MES
MON
NIC
SAR
Individuals

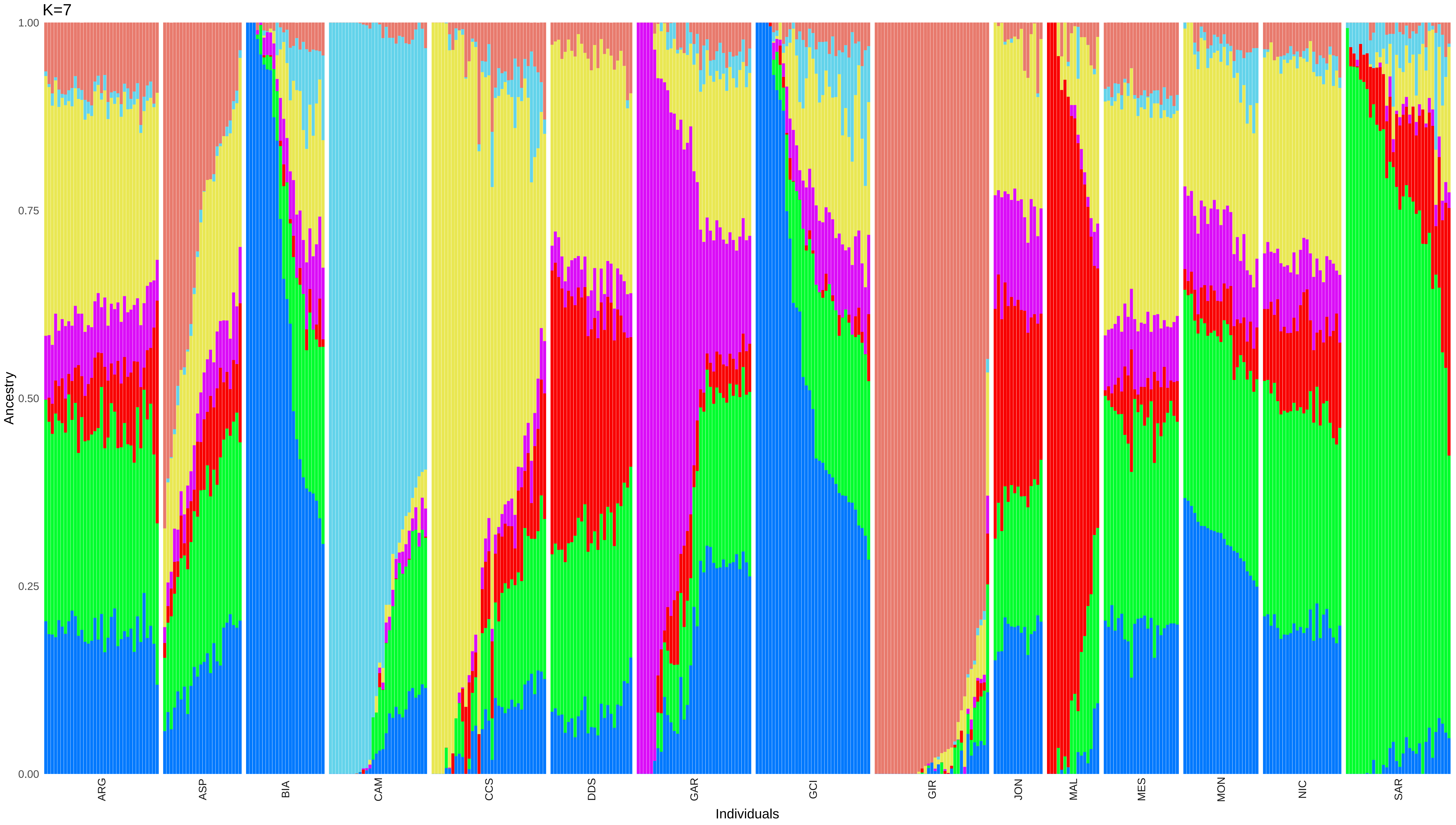
K=7
1.00
0.75
0.50
0.25
0.00
Ancestry
ARG
ASP
BIA
CAM
CCS
DDS
GAR
GCI
GIR
JON
MAL
MES
MON
NIC
SAR
Individuals

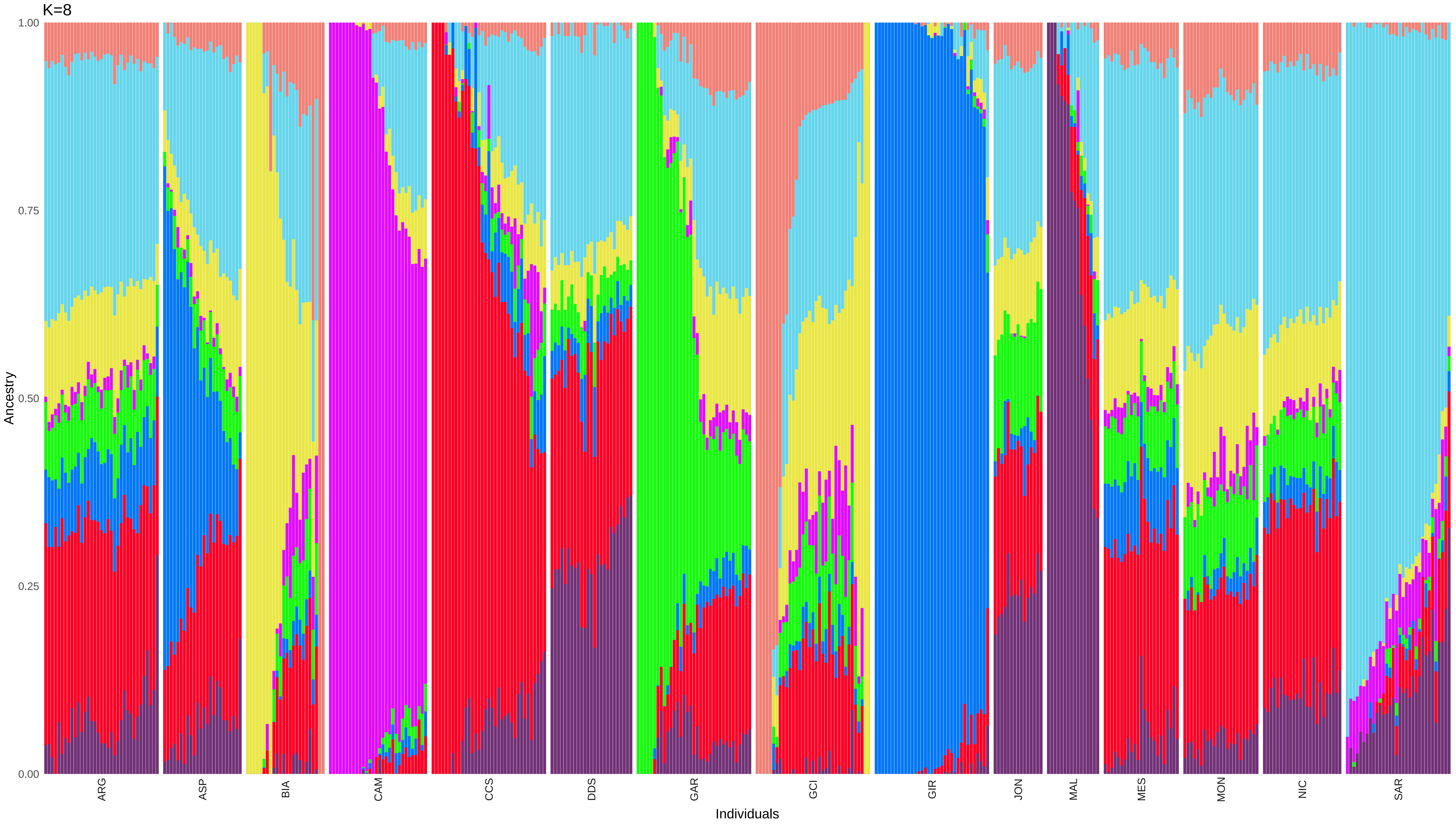

K=8
1.00
0.75
0.50
0.25
0.00
Ancestry
ARG
ASP
BIA
CAM
CCS
DDS
GAR
GCI
GIR
JON
MAL
MES
MON
NIC
SAR
Individuals

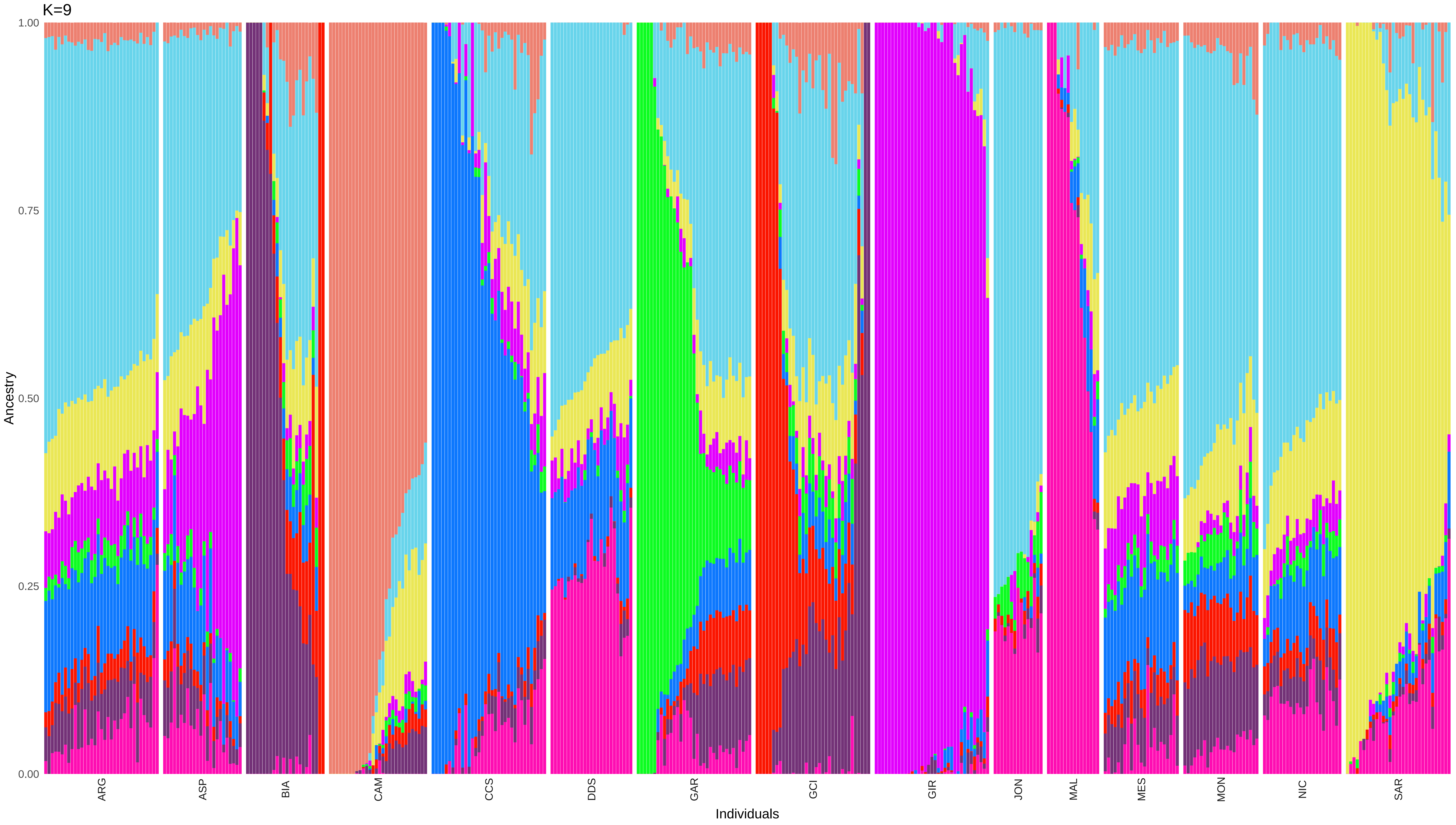
K=9
1.00
0.75
0.50
0.25
0.00
Ancestry
ARG
ASP
BIA
CAM
CCS
DDS
GAR
GCI
GIR
JON
MAL
MES
MON
NIC
SAR
Individuals

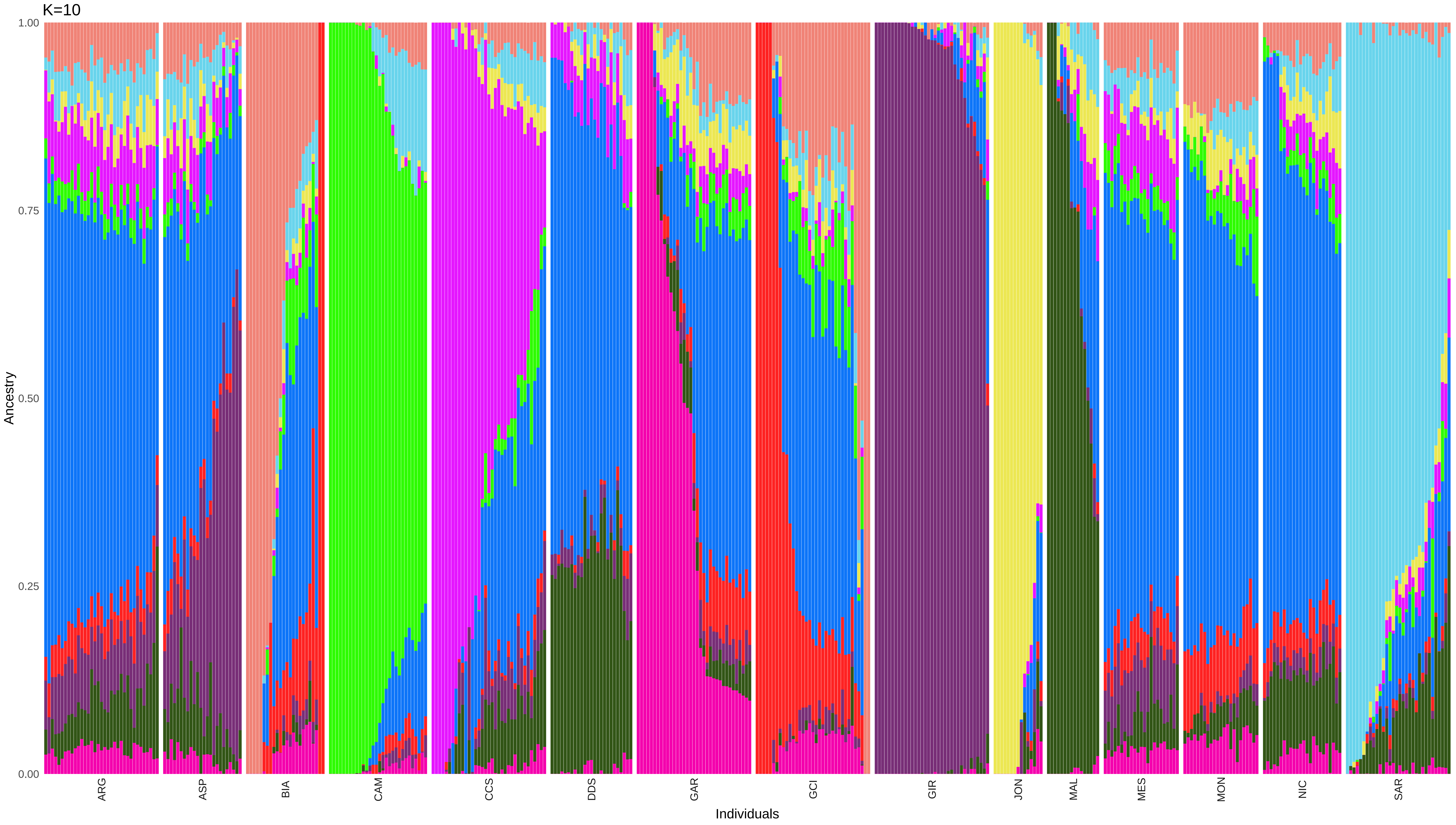
K=10
Ancestry
1.00
0.75
0.50
0.25
0.00
ARG
ASP
BIA
CAM
CCS
DDS
GAR
GCI
GIR
JON
MAL
MES
MON
NIC
SAR
Individuals

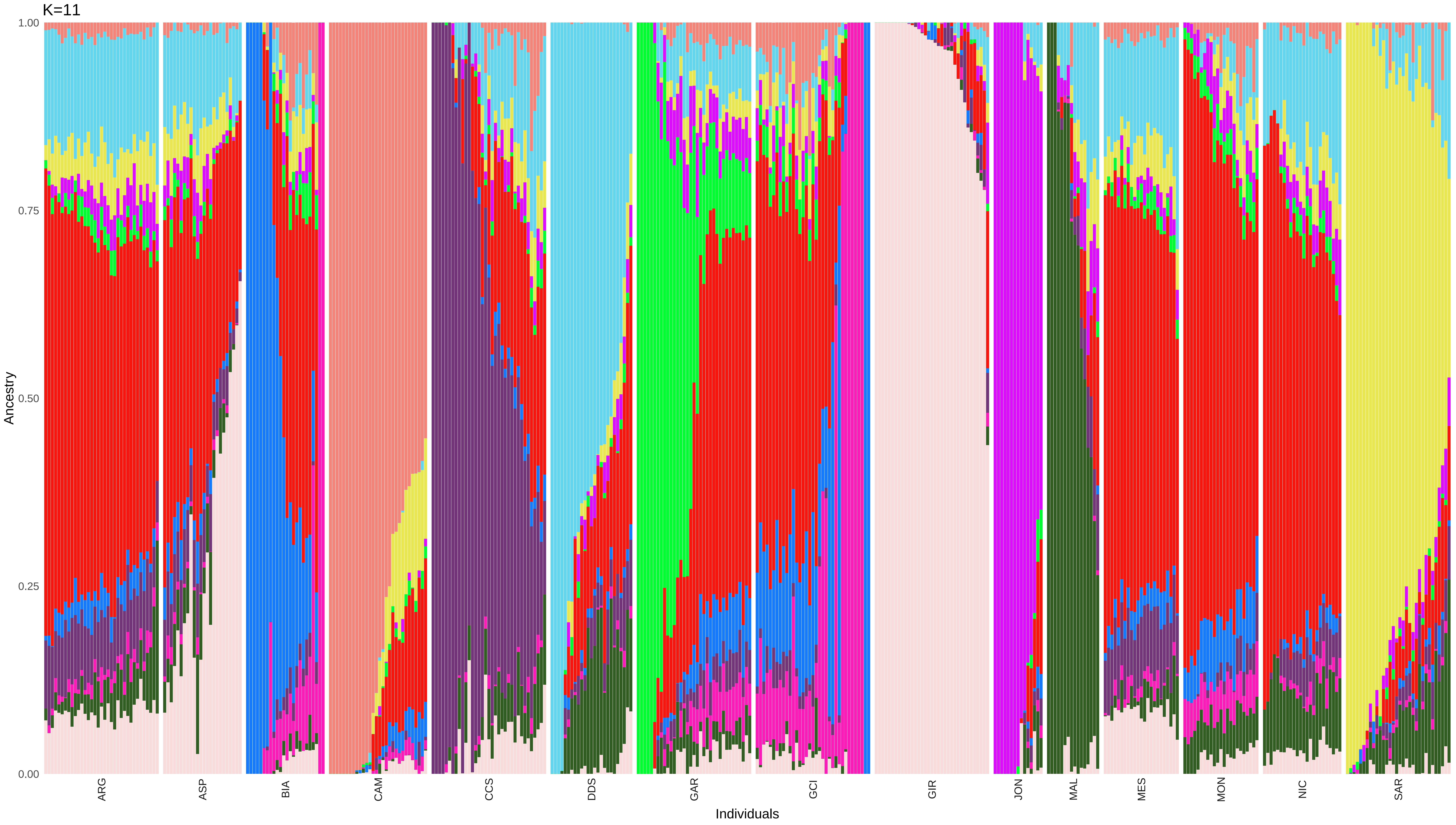
K=11
Ancestry
1.00
0.75
0.50
0.25
0.00
ARG
ASP
BIA
CAM
CCS
DDS
GAR
GCI
GIR
JON
MAL
MES
MON
NIC
SAR
Individuals

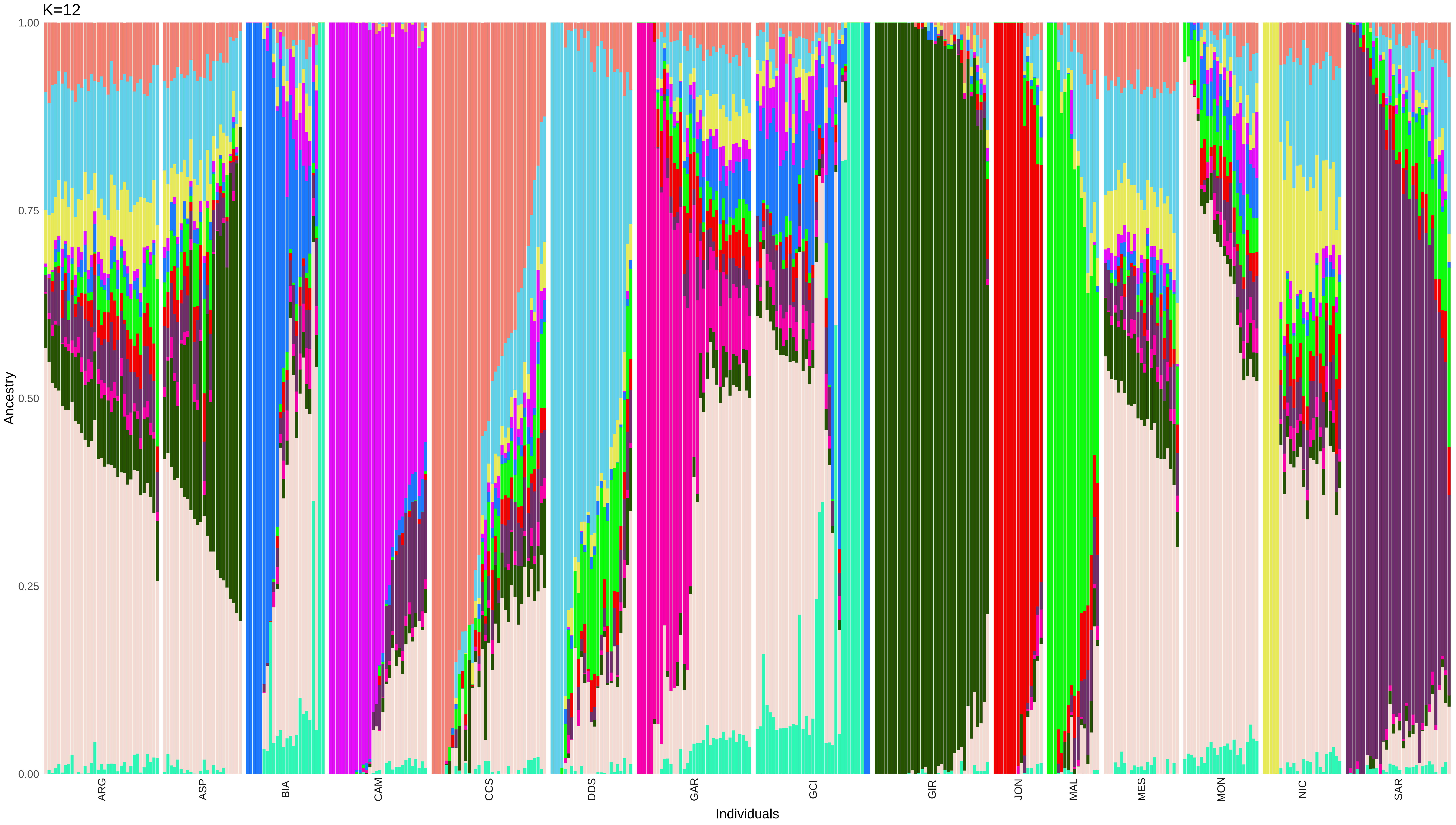
K=12
Ancestry
1.00
0.75
0.50
0.25
0.00
ARG
ASP
BIA
CAM
CCS
DDS
GAR
GCI
GIR
JON
MAL
MES
MON
NIC
SAR
Individuals

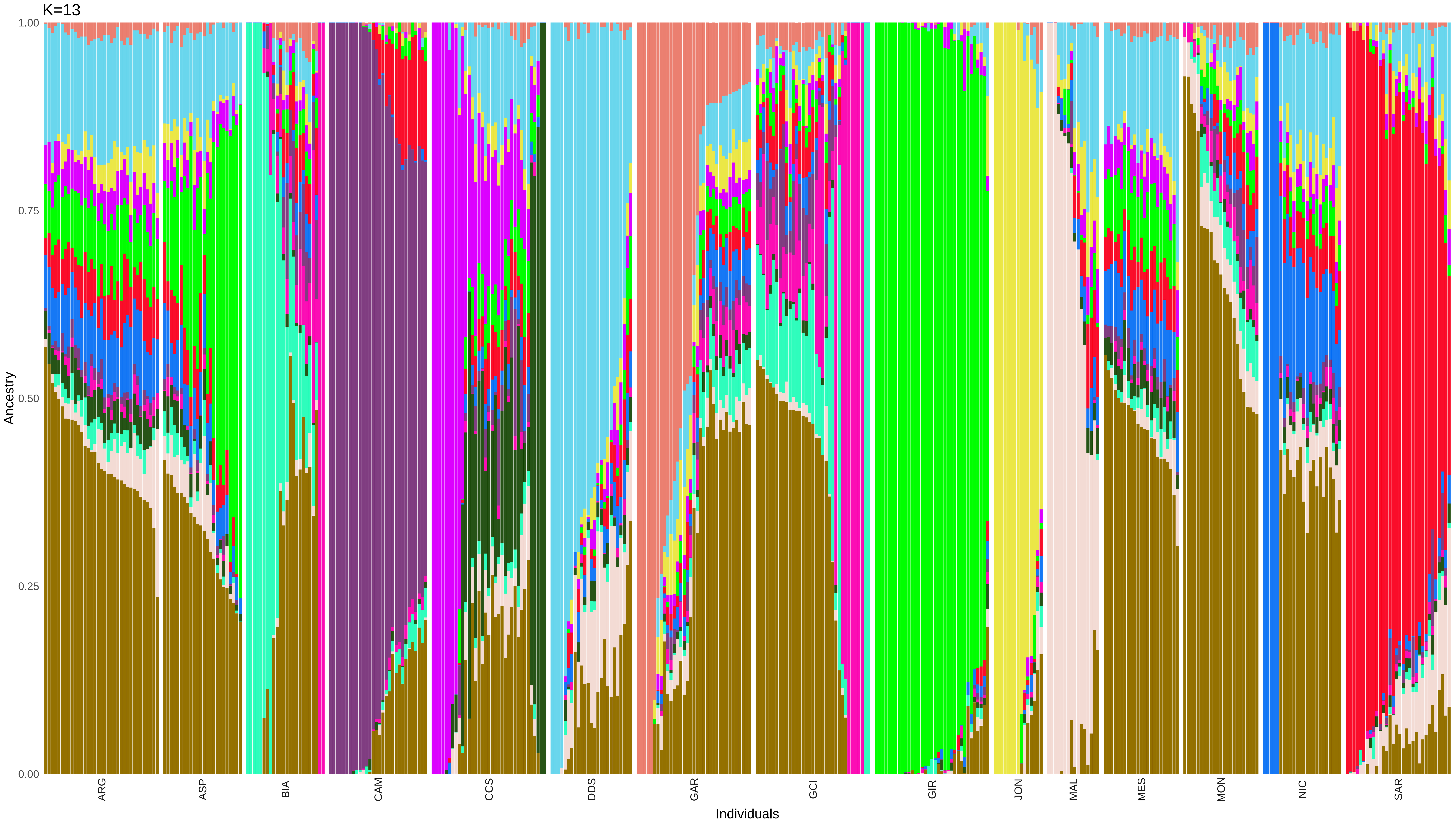

K=13
Ancestry
1.00
0.75
0.50
0.25
0.00
ARG
ASP
BIA
CAM
CCS
DDS
GAR
GCI
GIR
JON
MAL
MES
MON
NIC
SAR
Individuals

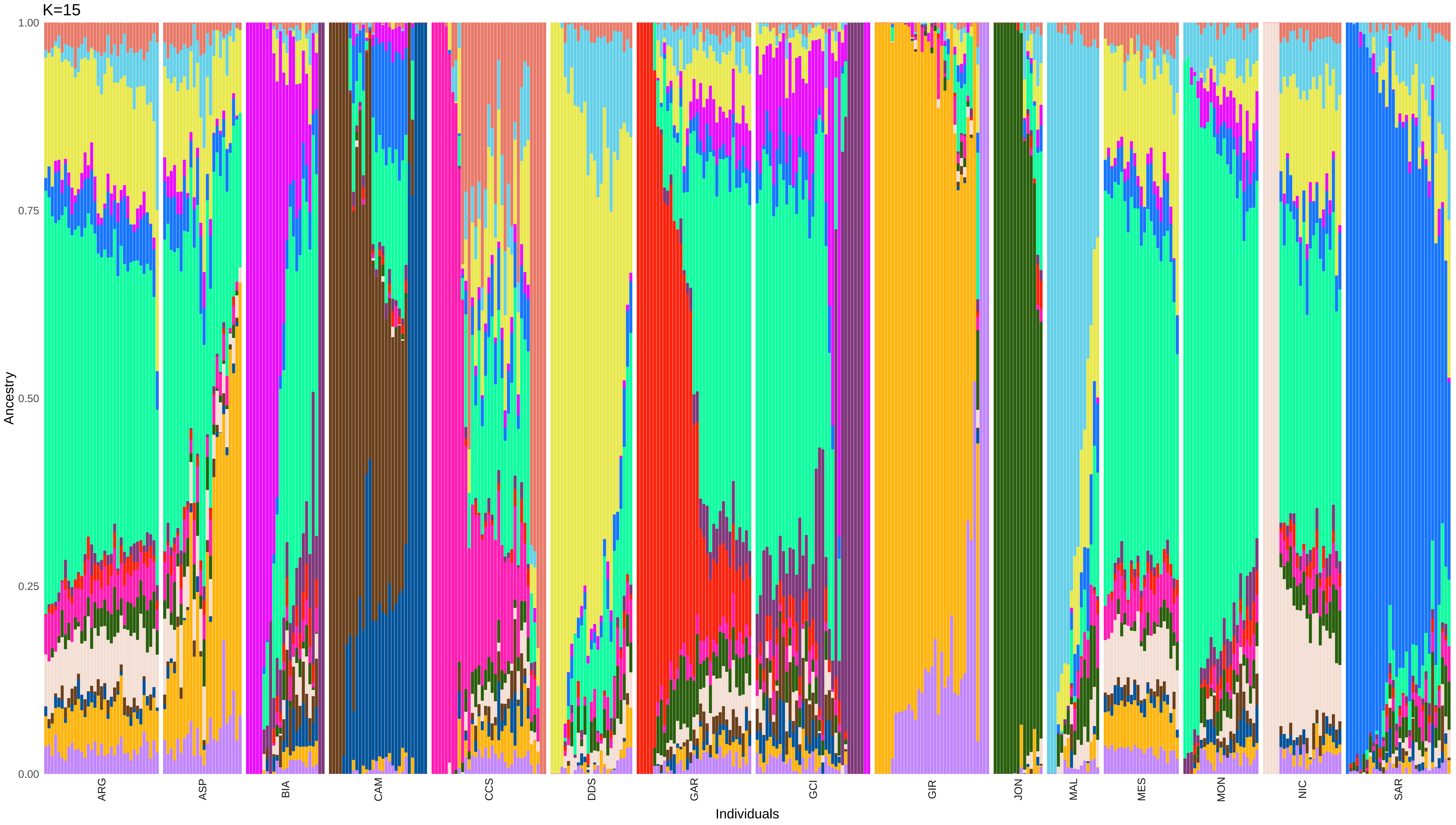
K=15
Ancestry
1.00
0.75
0.50
0.25
0.00
ARG
ASP
BIA
CAM
CCS
DDS
GAR
GCI
GIR
JON
MAL
MES
MON
NIC
SAR
Individuals

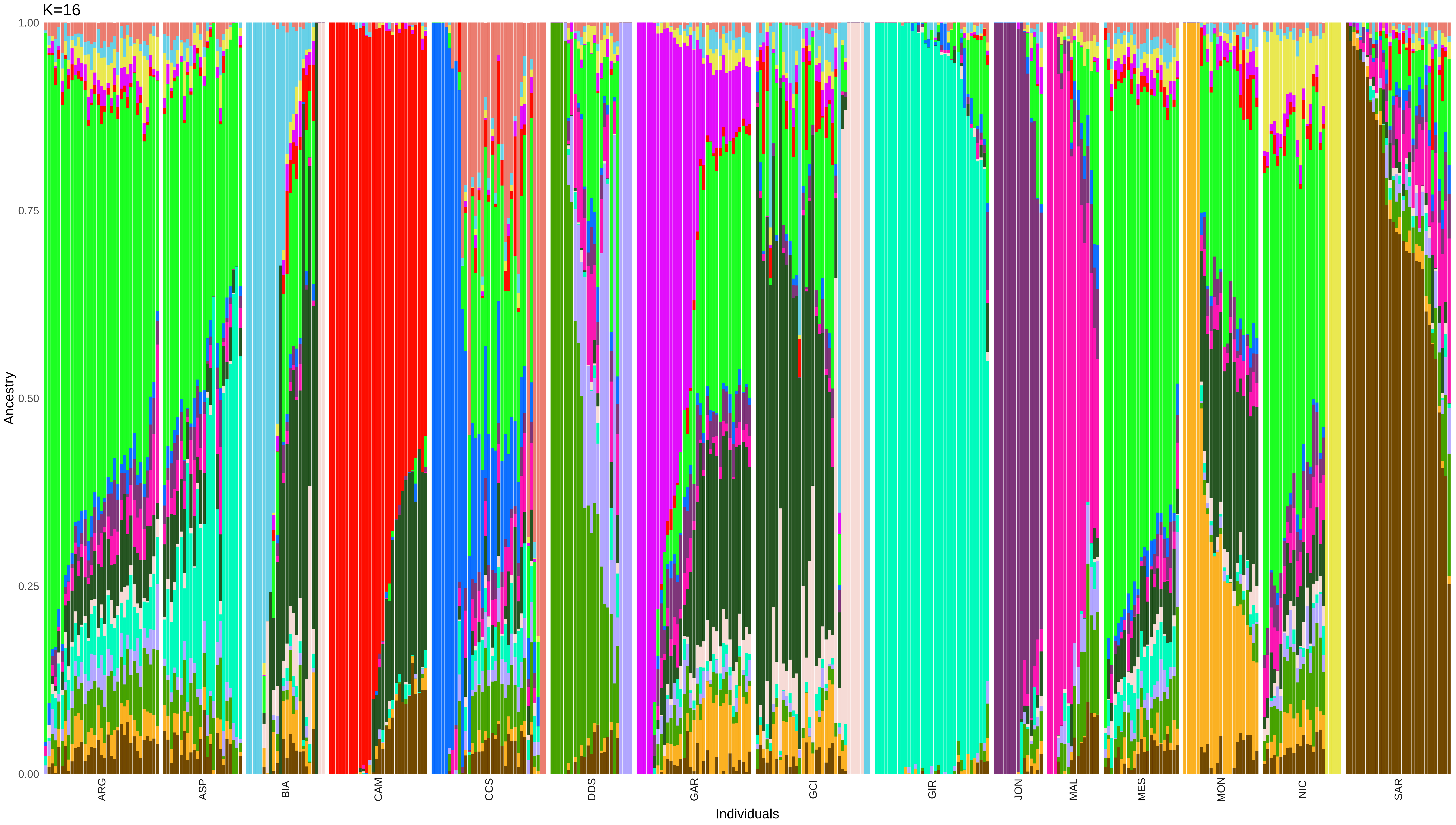
K=16
1.00
0.75
0.50
0.25
0.00
Ancestry
ARG
ASP
BIA
CAM
CCS
DDS
GAR
GCI
GIR
JON
MAL
MES
MON
NIC
SAR
Individuals
