# Supplementary material for: Phenotypic and Genomic Characterization of the Comune di Sicilia Goat: Towards the Conservation of an Endangered Local Breed
Source: Animals (Basel). 2023 Oct 13;13(20):3207. doi: 10.3390/ani13203207 (PMC10603724; doi:10.3390/ani13203207)
Supplement: Supplementary file 1 [file animals-13-03207-s001.zip › Table S1.pdf]

Table S1. Descriptive statistics of morphological traits in adult male Comune di Sicilia goats.

Morphological trait	Mean	Quartiles Q1	Median	Q3	SD	95% CI	Skewness	Kurtosis	*p*-value [1] Shapiro-Wilk
Body weight (BW)	53.7	42.3	52.3	65.2	17.54	25.82–81.64	0.46	1.47	0.81
Hearth girth (HG)	88.1	80.7	88.0	95.5	11.26	70.21–106.0	0.07	1.34	0.86
Croup height (CrH)	75.0	72.2	75.0	77.7	3.19	69.93–80.07	0	-5.76	0.11
Chest height (ChH)	36.7	35.5	35.7	38.0	2.18	33.28–40.22	1.93	3.75	<0.05
Wither height (WH)	74.7	72.2	74.7	77.2	3.23	69.61–79.89	0	-1.2	0.97
Chest length (ChL)	42.9	38.2	43.0	47.5	5.40	34.30–51.45	-0.04	-5.45	0.19
Trunk length (TL)	75.4	72.0	74.0	77.7	4.19	68.71–82.04	1.69	3.29	0.12
Croup length (CrL)	27.7	26.3	27.4	29.0	1.77	24.87–30.50	0.71	0.04	0.86
Chest width (CW)	21.3	19.8	20.4	22.7	2.20	17.80–24.80	1.66	2.64	0.10
Hip breadth (HB)	18.9	17.7	19.0	20.0	1.31	16.78–20.97	-0.12	-5.29	0.12
Coxo-femoral width (CxW)	21.8	20.7	21.5	23.0	1.58	19.34–24.36	1.07	0.65	0.61
Shin circumference (SC)	10.3	10.0	10.1	10.6	0.48	9.54–10.06	1.78	3.13	0.05

[1] Shapiro-Wilk test to assess if data are Normally distributed. The test compares the scores in the sample to a normally distributed set of scores with the same mean and standard deviation. $p < 0.05$ indicates that variable is not normally distributed.
