# Supplementary material for: Phenotypic and Genomic Characterization of the Comune di Sicilia Goat: Towards the Conservation of an Endangered Local Breed
Source: Animals (Basel). 2023 Oct 13;13(20):3207. doi: 10.3390/ani13203207 (PMC10603724; doi:10.3390/ani13203207)
Supplement: Supplementary file 1 [file animals-13-03207-s001.zip › Table S2.pdf]

Table S2. Comparison of morphological traits between subjects with or without horns, with or without wattles, and between farm 1 (Bolognetta farm) and farm 2 (Petralia farm) in adult female Comune di Sicilia goats.

	Farm		Horn		Wattle			P-value[2]		
Traits	1	2	No	Yes	No	Yes	SEM1	Farm	Horn	Wattle
Body weight (BW)	46.2	43.3	44.9	44.5	44.4	45.1	2.3	0.34	0.88	0.83
Hearth girth (HG)	83.7	81.5	82.7	82.5	82.2	82.9	1.6	0.31	0.94	0.76
Croup height (CrH)	66.9b	69.9a	68.7	68.1	69.1	67.7	0.9	0.01	0.55	0.24
Chest height (ChH)	35.4	34.9	35.2	35.1	35.6	34.7	0.5	0.52	0.91	0.22
Wither height (WH)	68.7b	71.2a	70.4	69.5	70.3	69.6	0.9	0.03	0.42	0.49
Chest lenght (ChL)	39.1	40.1	40.1	39.0	39.8	39.4	0.8	0.36	0.30	0.74
Trunk lenght (TL)	74.7	72.4	73.7	73.4	74.0	73.1	1.3	0.17	0.85	0.60
Croup lenght (CrL)	26.4	26.1	26.6	25.8	26.2	26.3	0.4	0.61	0.10	0.76
Chest width (CW)	21.9a	19.4b	20.8	20.5	20.5	20.8	0.4	0.01	0.47	0.49
Hip breadth (HB)	19.5	18.5	19.3	18.7	18.9	19.1	0.4	0.09	0.29	0.77
Coxo-femoral width (CxW)	21.0a	17.7b	20.2a	18.5b	18.9	19.8	0.6	0.01	0.04	0.24
Shin circumference (SC)	8.6	8.0	8.5	8.10	8.2	8.4	0.2	0.07	0.23	0.51

[1] Greatest standard error of the mean

[2] Horns: overall effect of horns (presence or absence); Wattles: overall effect of wattles (presence or absence); Farm: overall effect of Farm (1: Bolognetta farm; 2: Petralia farm).

[a-b] Different superscripts within a row and specific main effect (horn, wattle, or farm) indicate that means differ at P ≤ 0.05.
