# Supplementary figures and images for: Phenotypic and Genomic Characterization of the Comune di Sicilia Goat: Towards the Conservation of an Endangered Local Breed
Source: Animals (Basel). 2023 Oct 13;13(20):3207. doi: 10.3390/ani13203207 (PMC10603724; doi:10.3390/ani13203207)

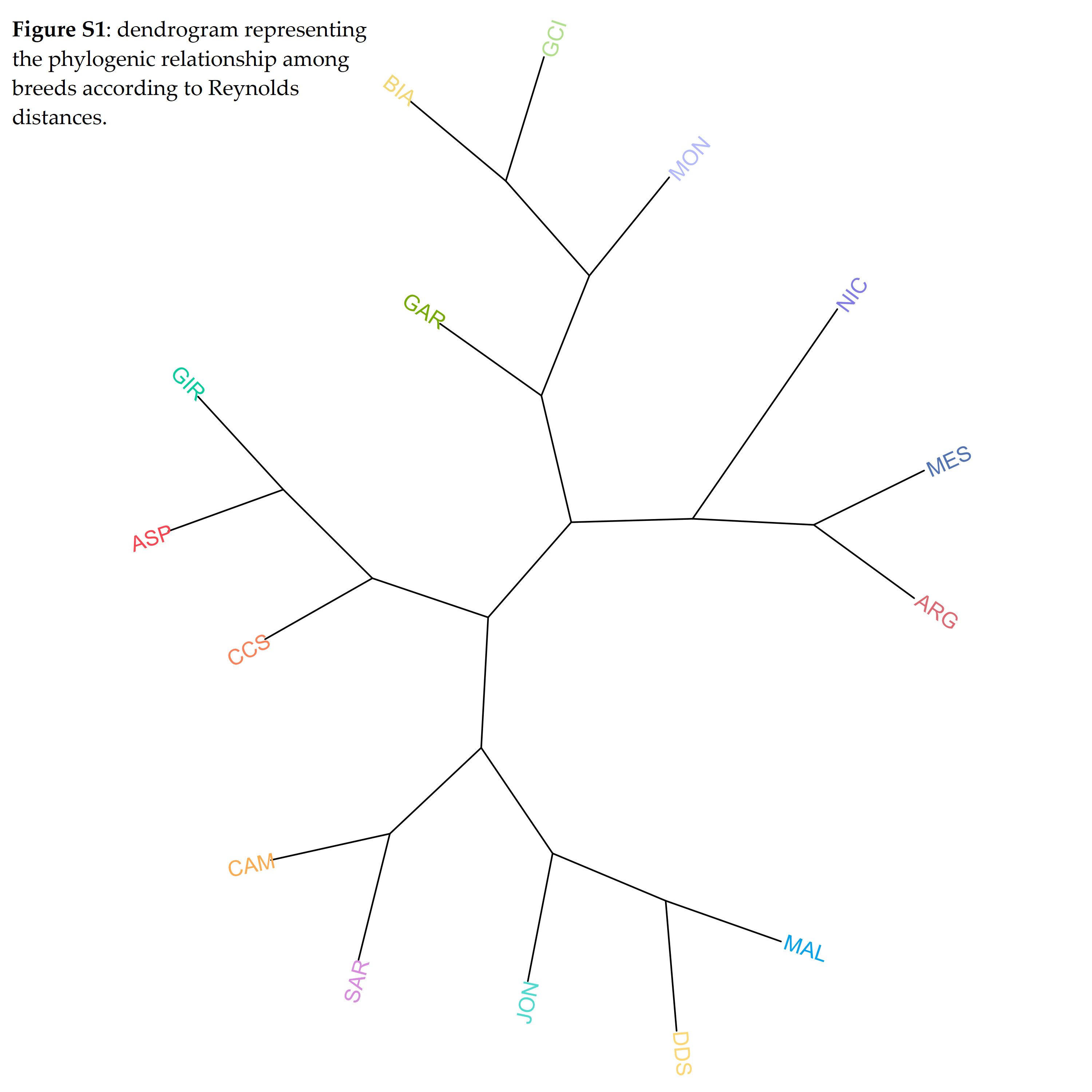

Supplement: Supplementary file 1 [file animals-13-03207-s001.zip › Figure S1.png]
